# Mutational signatures: the patterns of somatic mutations hidden in cancer genomes^☆^

**DOI:** 10.1016/j.gde.2013.11.014

**Published:** 2014-02

**Authors:** Ludmil B Alexandrov, Michael R Stratton

**Affiliations:** Cancer Genome Project, Wellcome Trust Sanger Institute, Wellcome Trust Genome Campus, Hinxton, Cambridgeshire CB10 1SA, United Kingdom

## Abstract

All cancers originate from a single cell that starts to behave abnormally due to the acquired somatic mutations in its genome. Until recently, the knowledge of the mutational processes that cause these somatic mutations has been very limited. Recent advances in sequencing technologies and the development of novel mathematical approaches have allowed deciphering the patterns of somatic mutations caused by different mutational processes. Here, we summarize our current understanding of mutational patterns and mutational signatures in light of both the somatic cell paradigm of cancer research and the recent developments in the field of cancer genomics.

**Current Opinion in Genetics & Development** 2014, **24**:52–60

This review comes from a themed issue on **Cancer genomics**

Edited by **David J Adams** and **Ultan McDermott**

For a complete overview see the Issue and the Editorial

Available online 29th December 2013

0959-437X/$ – see front matter, © 2013 The Authors. Published by Elsevier Ltd. All rights reserved.

**http://dx.doi.org/10.1016/j.gde.2013.11.014**

## Introduction

Long before the discovery of the double helix [1], it was well established that ultraviolet light (UV) can cause tumours of the skin [2]. While the mechanism was unclear at this time, it was hypothesized that successive doses of UV radiation result in accelerating the relative rate of cell proliferation [3]. The paradigm shifting discovery that the genetic material is contained within a deoxyribonucleic acid led to many studies in the late 1950s and throughout the 1960s examining how organisms protect their DNA from endogenous and exogenous mutations, and a focus was given to ultraviolet induced mutations (reviewed in Ref. [4^•^]). It was established that exposure to UV light can lead to the formation of dimers of any two adjacent pyrimidine bases on the same DNA strand with a preference for thymine–thymine dimers [4^•^]. It was further shown that UV irradiation damage predominantly results in cytosine to thymine or cytosine–cytosine to thymine–thymine changes, preferentially occurring at these pyrimidine dimers (i.e. C > T or CC > TT DNA mutations at dipyrimidine sites) [5,6]. This was the first detailed characterization of the pattern of DNA changes occurring due to the activity of an exogenous mutagen and, as such, the very first description of a signature of a mutational process.

While these early studies established the mutational signature of UV light, it was unclear whether UV induced mutations are present and involved in the neoplastic expansion of human cancers. The development of the DNA sequencing technique with chain-terminating inhibitors by Sanger *et al.* [7] allowed rapid examination of the genetic material contained in cancer cells. In the early 1990s, two studies sequenced exons of the gene *TP53* [8^•^,9^•^] from several patients and provided experimental evidence that aflatoxin and UV light leave distinct patterns (consistent with the ones observed in experimental systems) of DNA mutations respectively in hepatocellular and squamous-cell carcinomas. These studies confirmed that the mutational signatures of carcinogens are left as ‘evidence’ in the genomes of cancer cells [10] thus spawning research which first examined the mutations across *TP53* and later across multiple genes and even whole cancer genomes in order to provide a better understanding of the mutational processes involved in human carcinogenesis.

## Mutational patterns of *TP53*

Multiple independent studies used Sanger sequencing of some (or all) exons of a cancer gene to provide clues to the aetiology of both endogenous and exogenous factors of human carcinogenesis. *TP53* was usually selected for this analysis due to its high prevalence of somatic mutations in almost all tumour classes [11^••^]. Commonly, each of these studies involved multiple samples of a cancer type that were examined for somatic mutations in *TP53* (studies reviewed in Refs. [11^••^,12,13]). The *TP53* somatic mutations were aggregated, their spectrum was reported as specific for the given cancer type, and this spectrum was then compared to mutations generated experimentally in *in vitro* or *in vivo* systems [11^••^,13]. It should be noted that the mutational spectra of other genes, albeit rarely, were also used for such analysis [14].

These early studies revealed a significant heterogeneity of the *TP53* spectra across different cancer types, which allowed associating some patterns of mutation to known carcinogens. Here, we provide a brief summary of some of the more important findings while details could be found in Refs. [11^••^,12,13]. The *TP53* spectrum of skin carcinomas exhibited C > T and CC > TT mutations at dipyrimidines (all substitutions and dinucleotide substitutions are referred to by the pyrimidine(s) of the mutated Watson-Crick base pair). This was consistent with the *in vitro* described mutational signature of UV light. The *TP53* mutational spectrum derived from lung cancers in tobacco smokers was overwhelmed by C > A substitutions, which coincided with the class of mutation produced experimentally as a result of bulky adduct formation by tobacco carcinogens on guanine [15]. In other tobacco associated cancers, such as oesophageal and head and neck tumours, C > A mutations (while still ubiquitous) were less common while there was a significant increase of T > C mutations. Interestingly, in both smokers and non-smokers, C > T and C > G mutations at non-CpG sites were elevated when compared to all other cancer types, with bladder tumours harbouring the most C > G mutations [11^••^]. Additionally, it was demonstrated that C > A transversions were common in hepatocellular cancers and these mutations were believed to be associated with aflatoxin, a known carcinogen commonly found in food from southern Africa and Asia [16]. Lastly, all cancer types harboured at least some C > T mutations at CpG dinucleotides (mutated base underlined), a process attributed to the normal cellular event of deamination of 5-methylcytosine [11^••^].

The analyses of *TP53* spectra were the first attempts to bridge the gap between molecular cancer genetics and epidemiology [17]. The large number of studies examining *TP53* spectra required a computational resource to facilitate and retrieve the already identified somatic mutations. At first these data were managed by the researchers that were generating it but in 1994 the International Agency for Research on Cancer (IARC) started to maintain a database while providing a free access to it [17]. The first release of the IARC *TP53* database contained ∼3 000 somatic mutations [18] while the most recent version (R16) released in November of 2012, which can be found at http://p53.iarc.fr/, contains almost 30 000 somatic mutations in *TP53*.

Though extremely informative, the data gathered from single gene studies have significant limitations. In these studies, the spectrum of a cancer type is reported by aggregating mutations from multiple samples. This may be adequate when a single mutational process generates the majority of mutations in the particular cancer (e.g. UV light is the predominant mutational process in melanoma [19^••^]). However, usually multiple mutational processes are operative in a single cancer sample, and combining their mutations generates a mixed composition of the patterns of somatic mutations. In most cases, reporting this jumbled spectrum is uninformative for the diversity of mutational processes operative in a single cancer type or in a single cancer sample [20^••^]. Moreover, the examined *TP53* exons are both under selection and also have a specific nucleotide sequence. This affects the opportunity for observing a somatic mutation and as such the reported spectrum can be a reflection of the processes of selection and/or the nucleotide architecture of the *TP53* gene in addition to the processes of mutation [21,22].

Two studies tried to overcome some of the single gene limitations by leveraging a targeted capillary sequencing approach of large number of genes. A survey of the 518 protein kinase genes in 25 human breast cancer samples revealed 92 somatic mutations (90 substitutions and 2 indels) in which C > T transitions and C > G transversions preceded by thymine (i.e. C > T and C > G at TpC, mutated base is underlined) occurred with a higher than expected frequency [23]. This survey was later expanded to 210 cancer samples and it revealed more than 1 000 somatic mutations with significant variations in their patterns across the examined twelve cancer types [24]. Only a small fraction of the mutations reported in these screens are likely to be affected by selection [25], thus indicating that the observed mutational patterns reflect the operative mutational processes in the analyzed samples and not the processes of negative or positive selection.

## Mutational patterns identified in next generation sequencing data

The development of second-generation sequencing technologies allowed examination of cancer exomes (i.e. the combined protein coding exons) and even whole cancer genomes. Sequencing cancer exomes has been generally preferred as the majority of known cancer-causing driver somatic substitutions, indels, and copy number changes (although generally not rearrangements) [21] are located in protein coding genes. As the nucleotide sequence of protein coding genes is ∼1% of the whole genome, analysis of exomes is considered an advantageous and cost effective methodology for discovering the genes involved in neoplastic development. As a result, many studies have focused predominantly on the generation and analysis of exome sequences [26].

Early next generation sequencing studies started revealing patterns of somatic substitutions in different cancer types. In 2010, two back-to-back studies in *Nature* reported the patterns of somatic mutations in a malignant melanoma [27^•^] and small cell lung carcinoma [28^•^]. As expected, a strong signature of tobacco carcinogens was found in the genome of the lung cancer, while the mutational signature of ultraviolet light overwhelmed the melanoma genome. These studies demonstrated the value of whole genome sequencing for evaluating signatures of mutational processes by providing greater resolution and mechanistic insight into mutational signatures due to known carcinogens, for example through the identification of a lower prevalence of mutations over the footprints of genes.

Multiple independent studies and international consortiums started sequencing large numbers of samples from both cancer genomes and exomes [26]. An integrated genomic characterization was reported for many different cancer types including: acute lymphoblast leukemia [29–31], acute myeloid leukemia [32], breast cancer [33^••^,34,35], chronic lymphocytic leukemia [36,37], colorectal cancer [38,39], oesophageal cancer [40], glioblastoma [41], cancers of the head and neck [42,43], kidney cancer [44–46], liver cancer [47,48], lung cancer [49–54], lymphomas [55,56], melanoma [57–60], multiple myeloma [61], ovarian cancer [62], pancreatic cancer [63,64], prostate cancer [65–68], stomach cancer [69–71], uterine cancer [72], and several different types of pediatric tumours [73–79]. While these studies focused on the identification of novel cancer genes, mutational spectra were usually reported for each of the examined samples and some studies even tried to associate certain types of somatic mutations with the activity of mutagens or the failure of DNA repair mechanisms. A brief summary of the mutational patterns identified in these cancer genomics studies is provided in the next paragraph.

In lung cancer, comparison between tobacco smokers and non-smokers revealed that smokers have on average 10-fold increase in the burden of somatic mutations in their cancer genomes [50,51]. Consistent with the experimental evidence for tobacco carcinogens, this elevation is mainly due to the increase of the number of C > A transversions [15]. Examination of the cancer genomes of melanomas confirmed that the majority of mutations are C > T and CC > TT at dipyrimidines in the ultraviolet-associated tumours, while acral melanomas exhibit predominantly C > T transitions at CpG sites [59,60]. In glioblastoma multiforme, it was demonstrated that treatment with an alkylating agent, such as temozolomide, significantly elevates the numbers of somatic mutations and results in a distinct mutational pattern of C > T transitions [41]. In chronic lymphocytic leukemia, it was observed that samples with mutations in the immunoglobulin genes have a higher proportion of T > G transversions [36]. This mutational pattern and its immediate sequencing context are consistent with the activity of the error-prone polymerase η during somatic hypermutation [36,80]. In endometrial and colorectal tumours, a set of ultra-hypermutators with increased mutational frequency of transversions was associated with somatic mutations in polymerase ɛ [44,72]. Microsatellite unstable gastric cancer were observed to have a higher mutation prevalence of both C > T transitions and C > A transversions [71]. Examining the cancer exomes of patients with urothelial carcinoma (of the upper urinary tract) revealed a large number of somatic mutations with an unique pattern of T > A transversions predominately located at CpTpG sites and possessing a very strong transcription strand bias [81]. This pattern of mutations was associated with exposure to aristolochic acid. In oesophageal cancer, a high prevalence of T > G transversions was observed [40] while certain breast cancer genomes were found to be overwhelmed with C > T and C > G mutations at TpC sites [35].

These next generation sequencing studies provided an unbiased look into the patterns of DNA changes across cancer genomes. While they resolved some of the previous limitations from *TP53* studies (mostly by examining large portions of the human genome which are usually not under selection and which have a nucleotide context that is representative of the whole human genome) they still did not address the important issue of examining mixtures of mutations generated by different mutational processes.

## Mutational signatures derived from patterns of somatic mutations

**Figure 1:**
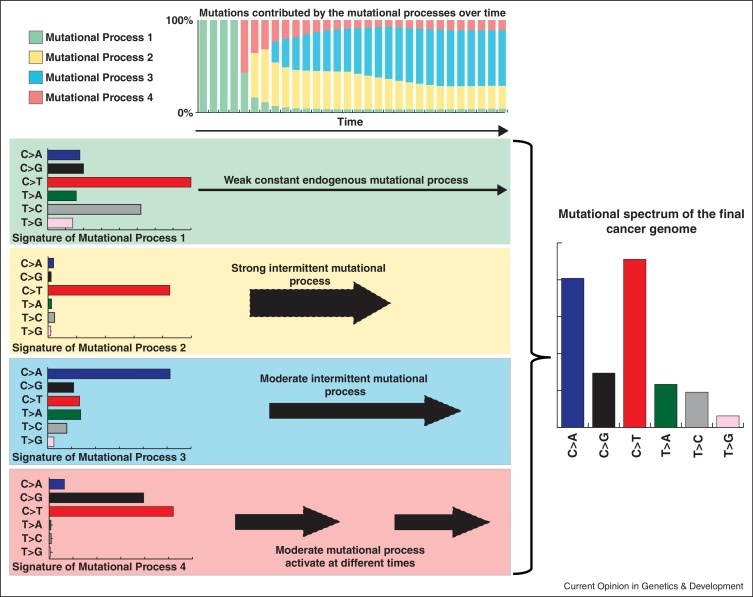
Mutational processes operative in a cancer. This simulated example illustrates four distinct mutational processes with variable strengths operative at different times throughout the lifetime of the patient. Each of these processes has a unique mutational signature exemplified by the six classes of somatic substitutions. At the beginning, all mutations in the cell (from which the cancer was eventually developed) were due to the activity of the endogenous mutational process 1. As time progresses, the other mutational process get activated and the spectrum of the cell continues to change. Note that the final sequenced cancer genome does not resemble any of the operative mutational signatures.

The somatic mutations in a cancer genome are the cumulative result of the mutational processes that have been operative since the very first division of the fertilized egg, from which the cancer cell was derived [21,22]. Each of these mutations was caused by the activity of endogenous and/or exogenous mutational processes with different strengths (Figure 1). Some of these processes have been active throughout the whole lifetime of the cancer patient while others have been sporadically triggered, for example, due to lifestyle choices (Figure 1). While examining patterns of somatic mutations can provide an indication of the aetiology of the operative mutational processes, it does not allow deciphering the individual mutational signatures that are operative in each sample as usually the pattern of a sequenced cancer genome does not resemble any of the operative mutational processes (Figure 1).

Recently, a theoretical model and computational framework that allows decomposing distinct patterns of somatic mutations from a set of cancer samples was developed [20^••^]. The mathematical model was an extension of the well-known blind source separation problem, in which original signals need to be separated from a set of mixed signals [82], and the algorithm was based on a method used in face recognition software that allows meaningfully learning distinct parts of objects [83]. The algorithm deciphers the minimal set of mutational signatures that optimally explains the proportion of each mutation type found in each cancer sample and then the method estimates the contribution of each signature to each cancer sample (see Ref. [20^••^] for more details about this method, including a discussion of its limitations).

Initial application of this approach was performed on the somatic substitutions derived from the whole genomes of 21 breast cancer patients [33^••^]. In order to increase the resolution of the derived mutational signatures, substitutions were examined using their immediate sequencing context. This included the base immediately 5′ before the somatic mutation and the base immediately 3′ after the somatic mutation; thus resulting in 96 mutation types — 16 different for each of the six types of somatic substitutions. For example, C > T mutations were extended to include C > T with (5′ adenine): ApCpA, ApCpC, ApCpG, ApCpT; (5′ cytosine): CpCpA, CpCpC, CpCpG, CpCpT; (5′ guanine): GpCpA, GpCpC, GpCpG, GpCpT; and (5′ thymine): TpCpA, TpCpC, TpCpG, TpCpT. Including the immediate sequence context allows better differentiation between different mutational processes; for example, distinguishing between C > T mutations due to the formation UV-light induced photodimers (i.e. C > T mutations at dipyrimidine sites such as TpCpC or CpCpC) from C > T mutations due to deamination of 5-methylcytosine (i.e. C > T mutations at CpG sites). The mutational catalogues of the 21 breast cancer genomes were generated, including each of the 96 mutation types, and applying the newly developed method to these catalogues revealed multiple distinct mutational signatures of substitutions. As expected, a mutational signature with features of C > T mutations at CpG sites was identified in most samples, thus reflecting the activity of normal endogenous cellular processes. Further, a mutational signature with C > X mutations at TpC sites was identified and based on similarity between its mutational pattern and *in vivo* experimental data, it was proposed that this process is due to the activity of the APOBEC family of deaminases and more specifically APOBEC1, APOBEC3A, and/or APOBEC3B [84,85]. Additionally, a rather uniform mutational signature (no prominent features across trinucleotides) was also identified and, interestingly, the activity of this mutational signature in each of the 21 samples allowed separation (by unsupervised hierarchical clustering) of *BRCA1* and *BRCA2* wild-type breast tumours from *BRCA1* and *BRCA2* germline mutants. Another mutational signature with unknown aetiology and mutations predominately at C > G at TpC was also identified. In addition to these genome-wide signatures, a localized hypermutation (termed *kataegis*) was observed in some of the breast cancer samples. This localized hypermutation was predominantly constituted of C > T and C > G substitutions at TpC trinucleotides and it was speculated that it is also due to the activity of the APOBEC enzymes. Lastly, deciphering the independent mutational signatures operative in these breast cancer samples provided the means for timing their activity across different cancer subclones [86].

This initial analysis of the mutational signatures operative in the 21 breast cancer genomes revealed several intriguing mutational processes but its focus was predominantly on substitutions. However, the newly developed approach for deciphering mutational signatures also allows extending mutational signature analysis over an arbitrary selected set of biologically meaningful mutation types [20^••^]. To demonstrate its applicability, the mutational catalogues of the 21 breast cancer genomes were extended to include double nucleotide substitutions, indels at microhomologies, indels at mono/polynucleotide repeats, and even a complex mutation type such as *kataegis*. Reanalysing these mutational catalogues demonstrated that *kataegis* separates as its own mutational process. Further, double nucleotide substitutions and indels at microhomologies associated predominantly with the activity of the previously identified uniform mutational process. Lastly, indels at mono/polynucleotide repeats did not strongly associate with any of the previously described mutational processes [20^••^].

Extending the previously defined mutational catalogues illustrated the possibility of incorporating additional mutation types and it revealed some associations between substitutions and indels thus providing more biological insight into the identified mutational processes [20^••^]. Further biological insight was derived by analysing mutational catalogues that incorporate the transcriptional strand on which a substitution resides in the footprints of a gene. Thus, the previously defined 96 substitution types were extended to 192 mutation types. For example, the number of C > T mutations at TpCpA were split into two categories: the number of C > T mutations at TpCpA occurring on the untranscribed strand of a gene and the number of C > T mutations at TpCpA occurring on the transcribed strand. In general, one would expect that these two numbers are approximately the same unless the mutational processes are influenced by activity of the transcriptional machinery. This could happen, for example, due to recruitment of the transcription-coupled component of nucleotide excision repair (NER) [87^•^]. If a mutational process has a higher number of C > A substitutions on the transcribed strand compared to the C > A substitutions on the untranscribed strand (i.e. note that C > A mutations on the untranscribed strand is the same as G > T mutations on the transcribed strand), this could indicate that the mutations caused by this process are being repaired by NER. As such, this analysis provides a further insight into the operative mutational processes and their interaction with cellular repair processes. A known example of such strand bias due to interplay between a mutational process and a repair mechanism is the formation of photodimers due to UV-light exposure that are repaired by NER and result in a higher number of C > T mutations on the untranscribed strand [87^•^].

Analysing the transcriptional strand bias of the mutational signatures operative in the 21 breast genomes revealed a weak strand bias of C > A mutations with unknown aetiology [20^••^]. Interestingly, deciphering mutational signatures from 100 breast cancer exomes revealed exactly the same trinucleotide mutational signatures but with a different strand bias. Specifically, there was an elevation of C > X mutations at TpCpT on the transcribed strand of exomes, which was absent in the complete gene footprints derived from the 21 whole genome sequences [20^••^]. This transcriptional strand bias could be indicative of exon-specific repair processes that are active in the cell.

The extensive mutational signature analysis performed on the 21 breast cancer genomes was recently expanded and mutational signatures (including substitutions, indels, dinucleotide substitutions, *kataegis*, and strand bias) were deciphered from 30 different types of human cancer [19^••^]. The previously developed computational framework was applied to almost five million somatic mutations identified in 7 042 cancer samples (507 from whole genome and 6 535 from whole exome sequences). This included both previously published samples and newly sequenced whole genomes. The analysis revealed 27 distinct mutational signatures [19^••^]. 22 of these 27 mutational signatures were validated (i.e. confirmed by orthogonal technologies or other approaches), three were associated with technology-specific sequencing artefacts, and two of the mutational signatures remain un-validated due to the lack of access to the relevant biological samples.

This largest cancer genomics analysis to date provided the first global roadmap describing the signatures of mutational processes in human cancer. Each of the cancer types had at least two mutational signatures operative in it, while some (e.g. cancers of the liver and uterus) had up to six distinct mutational processes. Remarkably, most of the cancer samples had at least two mutational signatures active in them. Aetiology was proposed for 11 of the 22 validated mutational signatures. Two of the mutational signatures were associated with age of patient at cancer diagnosis and these signatures were present in 26 of the 30 cancer types and more than 70% of the samples. These two processes exhibit clear features of C > T at CpG sites and most likely reflect mutations due to normal cellular processes (e.g. deamination of 5-methylcytosine, errors due to DNA replication, and so on) and probably account for the majority of somatic mutations prior to neoplastic development.

Based on similarity with *in vivo* experimental data, two mutational processes (termed Signature 2 and 13) were associated with the activity of the APOBEC family of deaminases. These two signatures exhibit predominantly C > T and C > G mutations at TpC sites and were observed in 16 of the 30 cancer types (∼17% of all examined cancer samples) [19^••^]. As such, the activity of these mutational signatures (and respectively the APOBEC enzymes) is one of the most significant human carcinogens with prevalence superseding that of tobacco smoking and exposure to UV light. Recently, further evidence was provided for the involvement of APOBEC3B in human cancers, as its expression was elevated in tumours compared to their matched normal samples [88,89].

By comparing the substitution patterns of all signatures with experimental data, one of the mutational signatures was associated with exposure to ultraviolet light while another with benzo[a]pyrene, a known tobacco carcinogen. The signature associated with UV-light exhibited a higher presence of CC > TT dinucleotide substitutions as well as a strand bias indicative of the formation of photodimers, which further confirmed the association. In contrast, a mutational signature associated in lung cancer exhibited predominantly C > A mutations with a transcriptional strand bias suggesting the formation of bulky adducts on guanine. Interestingly, this mutational signature was also associated with CC > AA dinucleotide substitutions with a strong strand bias. Statistical tests comparing smokers with non-smokers in two cancer types (viz. lung adenocarcinoma and tumours of the head and neck) confirmed a highly significant elevation of this ‘tobacco smoking signature’ in smokers indicating that it was due to tobacco mutagens.

Further statistical analysis was performed to associate mutations in genes with the presence of mutational signatures. Distinct mutational signatures were associated with: mutations in BRCA1/2 in breast and pancreatic cancers; failure of the DNA mismatch repair pathway (e.g. due to methylation of the MLH1 promoter) in colorectal cancers; hypermutation of the immunoglobulin gene in CLL; recurring polymerase ɛ mutations in uterine and colorectal cancers. Interestingly, the mutational signature associated with failure of DNA mismatch repair was observed in nine different cancer types. While this process was operative in ∼20% of colorectal cancers and ∼15% of uterine cancers, it was also found in at least 1% of cancer samples in another seven cancer types. Another interesting observation was that while almost all BRCA1/2 mutants exhibit a specific mutational signature, there were also BRCA1/2 wild-type samples with high number of mutations due to this mutational process. Thus, it is possible that some BRCA1/2 wild-type samples might harbour somatic mutations or other abnormalities that result in a failure of homologous repair and activation of this mutational process.

Chemotherapy treatment could cause its own set of somatic mutations [24]. Examining the pre-treatment history of all 7 042 cancer samples revealed that melanomas and glioblastomas pre-treated with an alkylating agent exhibit a distinct mutational signature.

The performed global analysis was able to propose an association for 11 of the 22 validated mutation signatures, while the origins and aetiology of the other 11 mutational signatures remains unknown. Lastly, this study also examined the presence of loci of *kataegis* across human cancer and it revealed that *kataegis* is not confined only to breast cancer but it is also present in at least another seven cancer types including pancreas, lung, liver, medulloblastoma, CLL, B-cell lymphomas, and ALL.

## Conclusions and future promises

In the past five decades, analysis of mutational patterns has evolved from *in vitro* observation of DNA changes caused by ultraviolet light, to examination of the mutational spectra generated by sequencing single cancer genes in multiple samples, to performing targeted capillary sequencing screens of multiple genes across hundreds of samples, and more recently to large-scale analysis of the genomes of thousands of cancer patients revealing the signatures of the mutational processes involved in the development of their tumours. In the next decade, thousands of new whole cancer genomes across the majority of cancer types [26] will be generated, which will allow the creation of a final and comprehensive map of mutational signatures. The generation of such a mutagenesis map will most likely require the refinement of existing mathematical methods to accurately examine all known types of somatic mutations: substitutions, indels, copy number variations, structural rearrangements, and potentially even epigenetic changes. These analyses of next generation sequencing data must be complemented with experimental work revealing the aetiology of the identified mutational processes.

## References and recommended reading

Papers of particular interest, published within the period of review, have been highlighted as:

• of special interest
•• of outstanding interest
